# General cognitive performance declines with female age and is negatively related to fledging success in a wild bird

**DOI:** 10.1098/rspb.2022.1748

**Published:** 2022-12-21

**Authors:** Camilla Soravia, Benjamin J. Ashton, Alex Thornton, Amanda R. Ridley

**Affiliations:** ^1^ Centre for Evolutionary Biology, School of Biological Sciences, University of Western Australia, Perth, WA, Australia; ^2^ School of Natural Sciences, Macquarie University, Sydney, New South Wales, Australia; ^3^ Centre for Ecology and Conservation, University of Exeter, Penryn, UK; ^4^ FitzPatrick Institute of African Ornithology, University of Cape Town, Cape Town, South Africa

**Keywords:** cooperative breeding, southern pied babbler, general intelligence, cognitive senescence, sex differences, cognition

## Abstract

Identifying the causes and fitness consequences of intraspecific variation in cognitive performance is fundamental to understand how cognition evolves. Selection may act on different cognitive traits separately or jointly as part of the general cognitive performance (GCP) of the individual. To date, few studies have examined simultaneously whether individual cognitive performance covaries across different cognitive tasks, the relative importance of individual and social attributes in determining cognitive variation, and its fitness consequences in the wild. Here, we tested 38 wild southern pied babblers (*Turdoides bicolor*) on a cognitive test battery targeting associative learning, reversal learning and inhibitory control. We found that a single factor explained 59.5% of the variation in individual cognitive performance across tasks, suggestive of a general cognitive factor. GCP varied by age and sex; declining with age in females but not males. Older females also tended to produce a higher average number of fledglings per year compared to younger females. Analysing over 10 years of breeding data, we found that individuals with lower general cognitive performance produced more fledglings per year. Collectively, our findings support the existence of a trade-off between cognitive performance and reproductive success in a wild bird.

## 1. Introduction

The mental mechanisms through which animals acquire, process, store and act on information from the environment represent animal cognition [1]. Animals use cognitive mechanisms to adjust their behavioural responses to the environmental and social context, remember the location of resources, and learn which environmental cues indicate presence of food, mates or predators [2]. Different animal species rely on cognitive mechanisms to different extents to solve ecological problems (e.g. [3,4]), and even within a species, cognitive performance can vary significantly among individuals (e.g. [5,6]). Such great inter- and intraspecific variation has led to the question: what selective pressures shape the evolution of cognition?

Intraspecific studies of animal cognition have shown that in some cases cognitive performance is heritable [7]. Additionally, cognitive performance has been linked to mate choice [8], reproductive investment [6], reproductive success [5,9] and survival [10]. The existence of differential fitness arising from heritable variation in cognitive performance means cognitive traits can evolve [11]. However, better cognitive performance is not always associated with increased fitness, for example, faster learning is associated with reduced longevity in fruit flies (*Drosophila melanogaster*) [12]. Indeed, the energetic costs of enhanced cognitive function may lead to a trade-off with somatic maintenance or reproduction [11,13]. At the interspecific level, this energetic constraint may actually result in a positive relationship between longevity and cognitive performance: larger brains require a longer development, and as a consequence large-brained species often mature and reproduce later in life [13]. Therefore, to understand how selection acts on cognitive performance we need to consider its costs along with its benefits [13].

To understand how selection acts on cognition, the link between cognitive variation and fitness consequences needs to be identified, as well as the factors associated with individual variation in cognition. Several factors may explain differences in individual cognitive performance, including the physical and social environment [14,15], and individual attributes such as age (e.g. [16]), rank (e.g. [17]) and sex (e.g. [18]). Sex differences in cognitive performance often arise as a consequence of mating strategies or sex-specific ecological constraints [19]. For example, in the brood-parasitic brown headed cowbird (*Molothrus ater*), females outperform males on a large-scale spatial memory task, likely because the breeding strategy of this species relies on females finding host nests to lay their eggs in [20].

Age differences in cognitive performance have been mostly found when comparing juveniles and adults [21,22]. However, cognitive performance can also change during adult life [23,24]. The gradual reduction of cognitive function with age is known as cognitive senescence [25]. For example, homing pigeons (*Columba livia*) older than 10 years returned more often to feeders that they had just depleted despite them being empty, showing impaired short-term memory [16]. To date, little is known about cognitive senescence in the wild, due to the difficulties of quantifying individuals' age and testing cognition in the wild.

Growing evidence suggests that cognitive performance is related to social rank, but the direction of this relationship varies across studies [26,27]. Rank is determined based on social interactions, where dominant individuals monopolize resources [28]. It has been suggested that cognitive performance may not be related to dominance *per se*, but to covarying factors, such as vigilance, experience or motivation to find alternative food sources [17,27]. For example, in Arabian babblers (*Argya squamiceps*) subordinates were the first to learn to remove black lids in a novel foraging task, likely because they were more explorative, but dominants, which tend to be older in this species, were better able to generalize the solution to white lids because of experience [29].

Finally, individual cognitive performance can be influenced by group size because living in larger groups may require better cognitive performance in order to monitor the state and actions of group members, remember their identity, and the outcome of past interactions [5,14]. Despite the growing number of studies investigating intraspecific differences in cognitive performance, these individual and social attributes (rank, sex, age and group size) have rarely been examined simultaneously while controlling for proxies of motivation, and evidence of their relative importance in driving cognitive variation is scarce.

If we identify what selection pressures drive variation in cognition, the question of whether these act on each cognitive trait separately, or jointly as part of general cognitive processes, remains. In humans, it has been repeatedly demonstrated that individual performance correlates positively across different cognitive tasks, and approximately 40% of the total variation in performance can be explained by a single general cognitive factor *g* [30]. This factor, also referred to as general intelligence or intelligence quotient (IQ), predicts important life outcomes, such as occupational attainment, health and longevity [30]. Recently, several studies in non-human animals have also described something akin to a general cognitive factor *g* explaining between 30% and 60% of variation in performance across a battery of cognitive tasks [31]. The evidence for *g* provided by animal studies, however, has encountered criticism. First, generating reliable measures of *g* in non-human animals requires robust psychometric test batteries targeting well-studied cognitive traits [32]. It is also worth noting that variation in the combination of cognitive tasks used in a test battery can lead to different estimates of *g* [33]. Second, results indicative of *g* may arise in the absence of a truly general cognitive factor if performance on different tasks is underpinned by the same cognitive mechanism—for instance, variation in associative learning performance could potentially impact performance across a range of tasks [34]. Therefore, the single factor extracted from animal cognitive test batteries does not necessarily equate to general intelligence or *g* as described in humans. Nonetheless, if performance measured across a battery of cognitive tasks can be explained by a single factor, hereafter referred to as ‘general cognitive performance (GCP)’ [5,35], and this factor predicts fitness in the wild [5], then it represents a measurable cognitive trait which may be under selection in animal populations [30].

Here, we tested wild adult southern pied babblers (*Turdoides bicolor*, hereafter ‘babblers’) on a psychometric test battery containing three tasks designed to quantify (i) associative learning, (ii) reversal learning, (iii) inhibitory control. These are well-studied cognitive traits that span different domains [32,36]. Additionally, they are likely to be ecologically relevant as they allow individuals to: learn predictive contingencies between environmental cues (associative learning); learn a new association when the previous one stops being rewarding (reversal learning) and control prepotent motor responses when counterproductive (inhibitory control) [36,37]. To achieve a comprehensive understanding of the relationship between different cognitive traits, the factors underpinning interindividual variation in cognition and the link between cognitive performance and fitness in a wild animal, we (i) tested whether individual cognitive performance was positively correlated across tasks and could be explained by a single factor (GCP); (ii) measured proxies of motivation and attributes of the individual and social group (age, sex, rank, group size) to identify determinants of individual cognitive performance and (iii) related individual cognitive performance to multiple measures of reproductive success.

## 2. Methods

### (a) Study site and species

Data were collected at the Kuruman River Reserve (26°58′ S, 21°49′E; South Africa, 33 km^2^) between September–March in 2018, 2019 and 2021 (fieldwork in 2020 was suspended due to the COVID-19 outbreak). The reserve is situated within the semi-arid Kalahari region, which is characterized by vegetated sand dunes. Pied babblers are medium-sized (60–90 g), sexually monomorphic passerines endemic to this region. They are cooperative breeders and live in groups, which include a dominant pair and subordinate helpers [38]. The dominant pair produces approximately 95% of the offspring [39,40] and pair bond tenure varies greatly (from less than 1 month to greater than 5 years) [41]. On average only 4% of subordinates live in non-natal groups each year [39]. Each group defends a territory of 50–80 hectares year-round [42].

The study population has been monitored since 2003 and is habituated to human presence [38], which allows researchers to present the birds with cognitive tasks. Ringing and blood sampling for molecular sexing are performed on nestlings 11 days post-hatching [43]. Therefore, each bird in the study population is identifiable by a unique ring combination, and sex and age are known for most adult birds. Adult immigrants are trapped with a walk-in trap for ringing and blood sampling. We considered immigrants to be at least 1 year old at the time they immigrated into our study population, and if they immigrated as dominants and bred on the first year in which they immigrated we considered them to be at least 2 years old, as dispersal and first breeding are rarely recorded before these ages, respectively [41,44]. On average, subordinate individuals are younger than dominants [44]. Rank (dominant versus subordinate) is easily inferred from aggressive displays by dominant individuals towards subordinates [38], affiliative behaviours between dominants [41], and overnight incubation by the dominant female [38]. During the study (2018–2021), the population comprised 14 groups (average group size 4 ± 1 s.d., range 2–7 adults). We tested different individuals each year: 13 individuals from six groups in 2018, 18 from 10 groups in 2019 and seven from four groups in 2021.

### (b) Cognitive test battery

The cognitive test battery consisted of three tasks designed to quantify (i) associative learning, (ii) reversal learning, (iii) inhibitory control. These cognitive tasks tapped into the natural terrestrial foraging behaviour of babblers [38], as they required them to peck downwards at a lid or move around a barrier on the ground to retrieve a food reward (a mealworm, *Tenebrio molitor* larva). The original cognitive test battery included a spatial memory task, but this was later excluded because individuals' behaviour when interacting with the task did not deviate from a random sampling strategy (see electronic supplementary material, section S3).

Cognitive testing was conducted between 05.00 and 19.00, when babblers were active. Cognitive tasks were always presented in the shade when the birds were not showing any heat dissipation behaviours (i.e. panting and wingspreading) to avoid potential confounding effects of heat stress [45]. All trials in a cognitive test were performed when the focal individual was out of sight of other group members. This meant starting testing when the focal individual was foraging apart from other group members (babblers often forage over 10 m apart from each other [46]) and repositioning the task between trials (each lasting less than 1 min) if a group member approached. The three cognitive tests were carried out at least 24 h apart and the order was randomized within individual, except for the reversal learning, which was always carried out the day after the associative learning test. Prior to quantifying learning performance, individuals were trained to peck the lids in a cognitive task to find the reward using unpainted lids (electronic supplementary material, section S1). In all tasks, if the focal bird did not interact with the task for 30 min, the test was paused and continued the following day; when this occurred, the count of correct and incorrect trials was carried over from one session to the following. If the passing criterion was not reached by 120 trials, the test was stopped and individual performance was recorded as 120 trials.

#### (i) Associative and reversal learning

**Figure 1.**
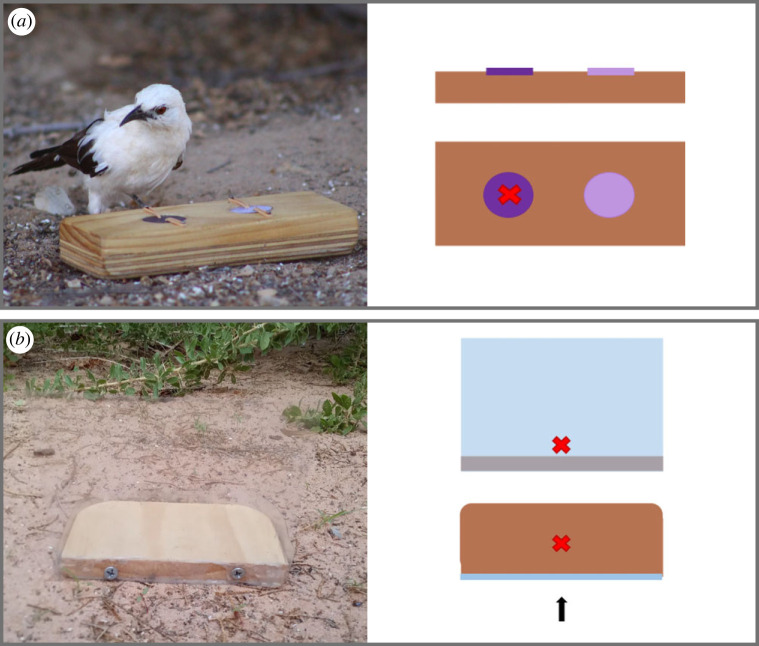
(*a*) Wild pied babbler interacting with the cognitive task used to quantify associative and reversal learning (left); and schematic of the associative and reversal learning task (right) showing both the overhead and front view. (*b*) Example of task used to quantify inhibitory control (left) and schematic of the inhibitory control task (right), which includes a frontal view and an overhead view with a black arrow indicating the direction of approach. The red X represents the location of the food reward: behind the barrier in the inhibitory control task and in the well of the rewarded colour shade (assigned randomly for each test subject) in the associative or reversal learning task. Photo credits: Nicholas Pattinson. (Online version in colour.)

The task used to quantify associative and reversal learning consisted of a small wooden block (180 × 70 × 30 mm) with two circular wells (30 mm diameter, 20 mm depth) covered by painted wooden lids. The lids were held in place by elastic bands; in this way, they fitted snugly into the wells, preventing the bird from using visual cues to identify the rewarded well, but they could swivel when pecked, making the food reward accessible (figure 1*a*). The two lids were painted a dark and light shade of the same colour rather than two different colours to avoid confounding effects of individual colour preferences due to past experience on learning performance (e.g. [35,47]; hereafter ‘colours’ instead of ‘colour shades’ for brevity). Each day before the start of cognitive testing, two mealworms were temporarily placed in both wells of the cognitive task to prevent the bird from relying on olfactory cues to choose the rewarded well during testing. The associative and reversal learning tests followed the protocols used by Shaw *et al*. [35] and Ashton *et al*. [5]. One of the two colours was randomly assigned to be the rewarded colour for each test bird. In each trial, the first peck of the individual when approaching the task was counted as correct (1 = rewarded lid) or incorrect (0 = unrewarded lid). During the first trial, we waited for all individuals to search both wells to see that only one hid the food reward, in subsequent trials, if the individual chose incorrectly the task was removed before the individual could gain the reward. The position of the rewarded well was pseudorandomized between trials to ensure the individual associated the colour and not the position of the lid with the reward. Associative learning performance was quantified as the number of trials required to reach the passing criterion, which was six correct choices in a row (a significant deviation from a random binomial probability: binomial test *p* = 0.016). If the bird passed the associative learning task, the reversal learning task was carried out 24 h after. Reversal learning performance was quantified using the same protocol and passing criterion used for associative learning, but rewarding the opposite colour.

#### (ii) Inhibitory control

We quantified inhibitory control using a detour-reaching task, which consisted of a transparent barrier (clear smooth PVC, 200 µm thick) fixed onto a wooden base, with a mealworm positioned approximately 2 cm behind the barrier on the wooden base (figure 1*b*). The task was presented to the individual straight on so that the mealworm was visible behind the barrier, but not accessible from the direction that the individual was approaching the task. In this task, the individual had to inhibit the prepotent response of pecking the barrier when seeing the food reward and instead detour around the barrier to retrieve it. A trial was marked as correct if the individual retrieved the mealworm without pecking the barrier. We did not include a training phase (see electronic supplementary material, section S1) but during the first trial we allowed the individual to eat the mealworm even if it pecked the barrier before detouring to make sure that the individual was aware of the presence of a barrier. The passing criterion was six correct trials in a row and the performance measure was the number of trials to criterion.

Detour-reaching tasks have recently been criticized because performance measures can be influenced by confounding factors, such as (i) past experience, (ii) body condition, (iii) neophobia or (iv) persistence [48]. However, our protocol allowed us to control for or test these factors: (i) none of the tested individuals had prior experience with a transparent barrier; (ii) we quantified individual body mass prior to cognitive testing; (iii) we quantified latency to approach the task, a proxy of neophobia, during testing (see §2c) and (iv) we measured performance as the number of trials taken to reach six correct detours around the barrier instead of the number of pecks to the barrier (the latter may be confounded by motivation and/or persistence).

#### (iii) Task variants

This study was conceived as part of a long-term project that involved repeatedly quantifying individual cognitive performance (see electronic supplementary material, section S7). To control for the potentially confounding effects of memory on cognitive performance, causally identical but visually distinct variants of each task (i.e. different colour combinations or shapes of the transparent barrier) were used for different replicates of the cognitive test battery over the course of the project [49]. The variant of the cognitive task used in a given replicate was assigned randomly to each individual. Task variant did not significantly affect the number of trials taken to pass the associative and reversal learning tasks nor the inhibitory control task (see electronic supplementary material, section S2). Therefore, in this study, we used individual cognitive performance measures obtained from the first replicate of the test battery, which comprised different task variants (e.g. associative learning might have been measured using dark/light green lids for one individual and dark/light purple lids for another).

### (c) Proxies of motivation

Performance in cognitive tasks may be influenced by the motivation of the individual to interact with the task [11]. When completing a cognitive task based on a food reward, individual performance might vary depending on hunger level and amount of food available in the environment. For this reason, we measured several proxies of motivation: foraging efficiency, body mass, latency to approach the task and inter-trial interval.

Weekly 20-min behavioural focal observations were carried out for all the individuals tested. Focal observations were conducted by continuously recording the behaviours of the individual (to the nearest second) using a customized program created in the free software Cybertracker. Foraging efficiency (grams of biomass consumed per foraging minute; following [50]) was calculated from focal observations comprising at least 5 min of foraging. As a previous study found babblers forage more efficiently in the early morning [51], we paired the timing of the focal observation and cognitive testing by performing both either in the early morning (before 09.00) or later in the day (after 09.00). Additionally, we measured the body mass of each individual (accuracy 0.1 g) within the 4 h prior to each cognitive test by enticing the individual to jump on a top-pan scale to retrieve a mealworm [52]. Finally, we measured the latency to approach the task as the time elapsed between the focal individual being within 5 m of the task and first making contact with the task [5], and the average inter-trial interval, which quantifies how quickly the individual approached the task again after a trial (electronic supplementary material, section S4).

### (d) Measures of reproductive success

Since 2003, each year during the breeding season (September–March) researchers perform weekly visits to the babbler groups during which the number and identity of individuals (adults, fledglings, juveniles) and any breeding activity are noted. Nests are located by observing nest building, and accurate hatch and fledge dates are recorded by checking the nests every 2–3 days. If fledglings are missing after two consecutive visits to the group, they are considered dead. We assumed only dominant individuals bred [39]; therefore, the offspring produced in each breeding attempt were attributed to the dominant male and female in the group at the time the attempt was recorded. The extensive life-history database allowed us to determine the number of fledglings produced, the number of fledglings surviving to independence (i.e. 90 days post-hatching, when offspring receive less than one feed per hour [52]), and the number of fledglings recruited (i.e. surviving to 1 year post-hatching [53]) per year for each dominant individual.

### (e) Statistical analyses

All analyses were performed with R statistical software v.4.2.0 [54]. We fitted different sets of generalized linear mixed models (GLMMs) using the *lmerTest* package [55] and tested the relative importance of different candidate explanatory terms by ranking them by Akaike information criterion corrected for small sample sizes (AICc). Models within two ΔAICc of the best model and with predictors whose 95% confidence intervals (CIs) did not intersect zero were included in the top model set, and were considered to explain variation in the dependent variable better than other candidate models [56]. Continuous predictors were scaled by centring on the mean and dividing by 1 s.d. Normality of residuals, presence of outliers and dispersion were checked using the *DHARMa* package [57]. To identify the minimum determinable effect of two-way interactions given our sample sizes [58], we conducted a power analysis with the *pwr* package [59].

#### (i) Relationships between individual cognitive performances across tasks

First, we tested whether cognitive performance was correlated across tasks by performing Spearman's rank correlations on the scores (i.e. number of trials to pass) of each pair of tasks. Note that a lower score in this case indicates fewer trials to pass the task, and hence, better cognitive performance. To determine whether individual performance in different tasks could be explained by a single factor (GCP), we then performed an unrotated principal component analysis (PCA) on the scores of the associative learning, reversal learning and inhibitory control tasks, using the *FactoMineR* package [60]. Following Shaw *et al*. [35], to test whether the mean and standard deviation of the loadings onto the first principal component (PC1) deviated from what is expected by chance, we performed 10 000 PCA simulations using the function *randomizeMatrix* in the *picante* package [61]. For each simulation, the cognitive scores within each task were randomized among individuals and a PCA was performed. We then compared the real mean and standard deviation of the loadings onto PC1 to the 95% CIs of the simulated means and standard deviations of the loadings onto PC1.

#### (ii) Factors explaining interindividual variation in cognitive performance

To determine whether individual and group attributes or proxies of motivation explained interindividual variation in cognitive performance, we fitted LMMs containing group identity as a random term and GCP as dependent variable, where GCP was the individual coordinate along PC1 but with the opposite sign so that higher values corresponded to higher GCP. We used GCP as a measure of individual cognitive performance because performance on all tasks loaded strongly and positively onto PC1 (§2e(i)). We considered as candidate explanatory terms: (i) individual and group attributes, i.e. age, sex, rank and group size; (ii) proxies of motivation, i.e. average latency to approach (s), inter-trial interval (min), body mass (g) and foraging efficiency (g min^–1^), all of which were averaged across the three tasks used to compute GCP; (iii) time of day; (iv) study year (2018, 2019 or 2021). We also included ‘testing order’ as a candidate explanatory term to confirm that individuals tested later within a group did not outperform those tested earlier (i.e. social learning was not occurring, *sensu* [5]). Overall, the model set included separate LMMs for each of the candidate explanatory terms listed above (i.e. age, sex, rank, group size, latency, inter-trial interval, body mass, foraging efficiency, testing order, time of day and study year) and for all possible pairwise additive models and pairwise interactions among sex, age, rank, group size, body mass and study year. Six individuals were excluded from this analysis due to unknown sex (molecular sexing not completed).

#### (iii) The relationship between cognitive performance and reproductive success

When analysing reproductive success, we considered only dominant individuals [39]. We included two individuals that were subordinates in the early years of testing but were retested once they gained dominance (total *N* = 19 dominant individuals). First, we checked whether the individual attributes that determine variation in cognitive performance, i.e. age and sex (based on the results of §2e(ii)), were also associated with variation in the average number of fledglings produced per year since 2 years of age, which is the earliest age at which individuals in our dataset bred (see electronic supplementary material, section S9). Hence, we determined if individual cognitive performance was directly related to reproductive success. We considered three measures of reproductive success: number of fledglings per year, number of independent offspring per year and number of offspring recruited per year. When there were multiple breeding attempts within a year we used cumulative numbers, and we assigned a 0 for years in which dominant individuals did not successfully breed. The average number of years with breeding data per dominant individual tested was 4.7 (range 1–11 years), where a year encompasses the austral breeding season (from September of one year to August of the next year). For each of the three measures of reproductive success we fitted a set of GLMMs with a Poisson error distribution and year and individual ID as random terms. Group ID was not included in these models as a random term because it resulted in overfitting (singular fit), but a parallel analysis including group ID instead of individual ID confirmed the results presented here. Each model set included separate GLMMs for GCP, age, sex, group size and drought (1 = drought versus 0 = no drought during the breeding season, where drought is defined as rainfall less than or equal to 137 mm [53]). We also included the interaction between GCP and sex to test if the relationship between cognition and reproduction differed in males and females.

## 3. Results

### (a) Relationships between individual cognitive performance across tasks

The 38 tested babblers completed the associative learning, reversal learning and inhibitory control tasks in a mean of 39.26 trials (range 6–120), 63.18 trials (range 6–120) and 34.58 trials (range 6–105), respectively. The range in the number of trials taken to pass the tasks indicates considerable intraspecific variation in cognitive performance (see electronic supplementary material, section S5 for a comparison with studies using similar test batteries on wild birds). We found positive correlations in cognitive performance for all pairwise comparisons across tasks, but only the correlation between associative and reversal learning performance was significant (associative and reversal learning: Spearman's rho = 0.65, *p* < 0.001; reversal learning and inhibitory control: Spearman's rho = 0.29, *p* = 0.08; associative learning and inhibitory control: Spearman's rho = 0.14, *p* = 0.42). The consistent positive direction of pairwise correlations between tasks aligns with the output of the PCA, which showed that all cognitive scores loaded positively onto PC1 extracted with an eigenvalue over 1 (table 1). PC1 explained 59.5% of the total variation in cognitive performance across tasks (table 1).

**Table 1.** Output of the principal component analysis on the scores (i.e. number of trials to pass) obtained by 38 pied babblers on three cognitive tasks quantifying associative learning, reversal learning and inhibitory control.

cognitive task	PC1
associative learning	0.82
reversal learning	0.88
inhibitory control	0.58
eigenvalue	1.79
% variance explained	59.54

The PCA results were highly unlikely to occur by chance because the real mean loading onto PC1 was higher than the 95% CI of the randomly simulated mean loadings (95% CI of simulated means for PC1 = 0.01–0.67, real mean = 0.76; electronic supplementary material, figure S3), and while the real s.d. was within the 95% CI of the simulated s.d., it was at the lower end of the distribution (95% CI of simulated s.d. for PC1 = 0.08–0.83, real s.d. = 0.16; electronic supplementary material, figure S3). Additionally, when we examined the cognitive scores of 18 individuals that were tested twice on the cognitive test battery during 2018–2021, individual scores from the second replicate of the cognitive test battery also loaded positively onto PC1, which explained 46.3% of the total variance in cognitive performance, providing further evidence for GCP; importantly, GCP was significantly repeatable (*R* = 0.50; s.e. = 0.18; 95% CI = 0.09; 0.78; *p* = 0.015) (see electronic supplementary material, section S7).

### (b) Factors explaining interindividual variation in cognitive performance

**Figure 2.**
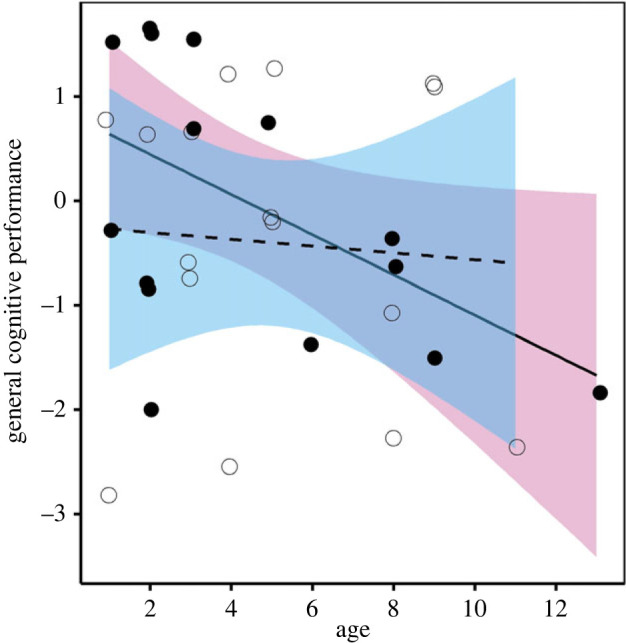
Variation in pied babblers' general cognitive performance by age and sex (females: pink colour, solid line, filled dots, *N* = 16; males: blue colour, dashed line, empty dots, *N* = 16). Points are raw data; fitted lines and 95% confidence bands are generated from the output of the model presented in table 2. (Online version in colour.)

The factors that best explained variation in GCP were age and sex (table 2). GCP declined with age in females but not males (females: coefficient ± s.e. = −0.77 ± 0.20, 95% CI = −1.18; −0.37, males: coefficient ± s.e. = 0.13 ± 0.24, 95% CI = −0.36; 0.62; *N* = 32, of which 16 females and 16 males; figure 2). Group size was not a significant predictor of GCP. Importantly, study year and the proxies of motivation examined (i.e. latency to approach, inter-trial interval, body mass, foraging efficiency, time of day) did not significantly explain variation in GCP (electronic supplementary material, table S3). The power analysis confirmed that we had enough power to detect moderate effects of main terms (Cohen's *f*^2^ = 0.26) and large effects of two-way interactions (Cohen's *f*^2^ = 0.39) given *N* = 32 individuals.

**Table 2.** Top model set of candidate terms affecting general cognitive performance in pied babblers. All models included group ID as a random term. AICc and ΔAICc are provided for models within 2 ΔAICc of the top model and with predictors whose 95% CI do not intersect zero. Coefficient estimates ± s.e. and 95% CI are given below the top model set. *N* = 32 individuals from 11 groups. See electronic supplementary material, table S3 for full model selection outputs.

top model set	AICc	ΔAICc
age × sex	103.53	0.00
*null model (intercept only)*	*109.46*	*5.93*
effect size of explanatory terms	estimate ± s.e.	95% CI
age	−0.76 ± 0.19	−1.14; −0.37
sex (male)	−0.48 ± 0.28	−1.05; 0.09
age × sex (male)	0.90 ± 0.29	0.29; 1.49

### (c) The relationship between general cognitive performance and reproductive success

**Figure 3.**
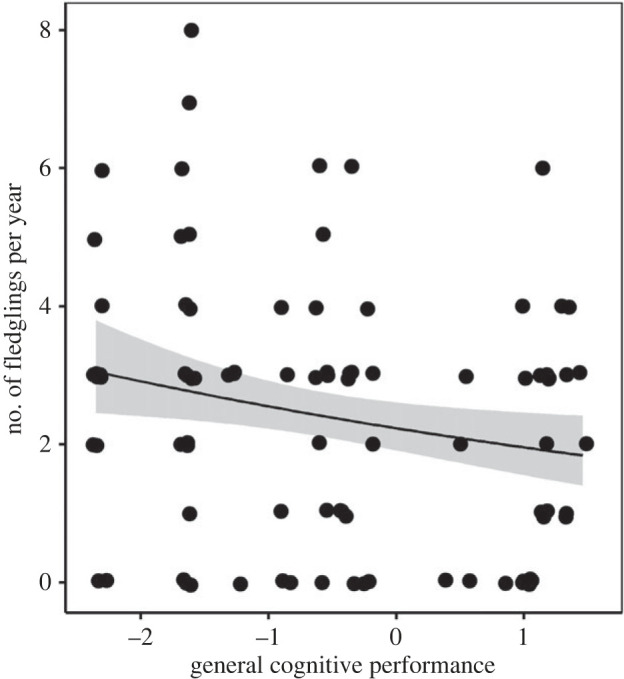
The relationship between the number of fledglings produced per year and general cognitive performance in dominant pied babblers (*N* = 90 observations for 19 dominant individuals over 14 years). Points are raw data; the fitted line and 95% confidence band are generated from the output of the model presented in table 3.

The average number of fledglings produced per year since age 2 tended to increase with age in females but not in males (electronic supplementary material, section S9). Hence, in females, the relationship between reproductive success and age followed an opposite trend compared to the relationship between GCP and age: older females tended to produce more fledglings per year on average but showed lower GCP. In line with this result, we found that individual GCP was negatively related with the number of fledglings produced per year (table 3*a*, figure 3). We did not find evidence that this relationship differed in males and females (non-significant interaction GCP × sex), but we only had power to detect very large effects of two-way interactions (Cohen's *f*^2^ = 0.75) given *N* = 19 individuals.

**Table 3.** Model set of candidate terms affecting three measures of reproductive success in pied babblers. The models included year and individual ID as random terms. Coefficient estimates ± s.e. and 95% CI are given below the model sets for models within 2 ΔAICc of the top model and with predictors whose 95% CI do not intersect zero. The measures of reproductive success examined over 14 years were: (a) number of fledglings produced per year, *N* = 90 observations for 19 dominant individuals; (b) number of fledglings reaching independence per year, *N* = 81 observations for 18 dominant individuals; (c) number of fledglings recruited per year, *N* = 79 observations for 14 dominant individuals.

model selection	AICc	ΔAICc
(a) number of fledglings per year		
GCP	356.08	0.00
GCP × sex^a^	357.38	1.30
sex^a^	357.46	1.38
age	358.76	2.68
*null model (intercept only)*	*359*.*01*	*2*.*93*
drought	359.75	3.67
group size	361.17	5.09
(*b*) number of independent offspring per year		
drought	258.70	0.00
*null model (intercept only)*	*264*.*59*	*5*.*89*
sex	264.80	6.10
age	266.09	7.39
GCP	266.26	7.56
group size	266.56	7.86
GCP × sex	269.21	10.51
(c) number of offspring recruited per year		
drought^a^	204.48	0.00
*null model (intercept only)*	*205*.*73*	*1*.*25*
age	205.77	1.29
sex	206.05	1.57
GCP	206.74	2.26
group size	207.80	3.32
GCP × sex	209.27	4.79
effect size of explanatory terms	estimate ± s.e.	95% CI
(a) number of fledglings per year		
GCP	−0.18 ± 0.07	−0.34; −0.03
(b) number of independent offspring per year		
drought	−0.99 ± 0.34	−1.82; −0.36

^a^Not included in the top model set because 95% CI intersect zero.

The main predictor of the number of fledglings surviving to independence was the occurrence of droughts, with more fledglings reaching nutritional independence in non-drought years (table 3*b*). None of the explanatory terms tested were a significant predictor of the number of fledglings surviving to recruitment (table 3*c*). However, we had to exclude the two dominant females who showed the highest GCP from the latter analysis due to missing data on the number of fledglings surviving to recruitment; therefore, the lack of an effect of GCP on the number of offspring recruited per year should be interpreted with caution.

## 4. Discussion

We quantified individual cognitive performance in a wild bird population to answer three central questions in cognitive ecology: (i) does performance co-vary across cognitive tasks, (ii) what drives these individual differences and (iii) is individual cognitive performance related to reproductive success.

Individuals differed greatly in their associative learning, reversal learning and inhibitory control performance. Those that learnt an association faster, were also faster at reversing the learnt association and showed better inhibitory control, as shown by positive (albeit not always significant) correlations in cognitive performance across tasks. Indeed, approximately 60% of the variance in individual cognitive performance across tasks could be explained by a single factor: GCP. Additionally, GCP was significantly repeatable (*R* = 0.50), indicating that our measure of GCP captured consistent interindividual differences in cognition. While we cannot completely exclude that motivation affected cognitive performance, we are confident that its effect on our measure of GCP was minimal because all the birds that were presented with the task interacted with it successfully and always ate the reward; more importantly, none of the measured proxies of motivation significantly explained variation in GCP. Therefore, our findings are consistent with the existence of a general cognitive factor underpinning performance across different cognitive domains in babblers. However, our test battery included only three cognitive tasks, which is the minimum number required to test for a general cognitive factor [30]. Therefore, future studies should consider expanding the test battery by including, for example, tasks assessing social cognition, the ability to make inferences and reaction time [31,35].

Alternative explanations for GCP are also possible. For example, the different tasks used may tap into the same cognitive process [34], so that they are effectively measuring the same cognitive trait instead of a common cognitive factor underpinning performance across domains. Therefore, whether statistical evidence for GCP indicates a truly general cognitive ability underlying performance across different cognitive domains remains to be determined.

We found no evidence that living in larger groups is linked to cognitive performance in pied babblers. This is in contrast to recent findings on Western Australian magpies (*Gymnorhina tibicen dorsalis*) [5]. The difference between the two species could depend on the degree to which their societies involve individualized relationships [62]. Babblers live in groups with high within-group relatedness and high reproductive skew [39,40]. By contrast, in Western Australian magpies, multiple individuals breed within the group, offspring care by helpers is facultative [63] and within-group relatedness is low [64]. These conditions give more scope for conflicts of interest and hence for a society where individuals may benefit from tracking the outcome of past interactions with other group members, negotiating access to resources and breeding opportunities, and engaging in strategic decision-making [65]. Therefore, rather than social living *per se*, we must consider the extent to which social systems present individuals with information-processing challenges, such as the need to navigate multiple differentiated social relationships [66]. To test this, future cognition studies should quantify the amount and diversity of social interactions among group members through social network analysis.

Individual variation in GCP was predicted by an interaction between age and sex, with cognitive performance declining with age in females but not in males. Faster cognitive ageing in females has been previously reported in humans [67], nematodes (*Caenorhabditis remanei*) [68], mice (*Mus musculus*) [69] and marmosets (*Callithrix jacchus*) [24]. However, the only study testing for cognitive senescence in the wild found no decline in spatial memory performance in mountain chickadees (*Poecile gambeli*) [25]. Hence, to our knowledge, our finding represents the first evidence of sex differences in age-related cognitive decline in a wild animal.

Senescence has been explained by two main evolutionary theories [70]. The ‘selection shadow’ theory states that selection strength decreases with age after sexual maturity [71]. Our data do not support this theory because babblers were still breeding up to 13 years of age, leaving ample opportunity for selection to act on cognitive traits among older individuals. A second theory is the life-history theory of ageing, which encompasses two convergent theories: the first states that due to the limited resources available to organisms, these must be traded-off between reproduction and somatic maintenance (disposable soma theory) [72]; the second states that alleles with beneficial effects early in life but detrimental effects later in life can be favoured by selection (antagonistic pleiotropy) [73]. Previous studies on babbler life history [44,74] provide some support for both these theories.

First, female babblers (but not males) engage in costly breeding competition [74,75]. Subordinate females compete both indirectly, by courting and nest building with unrelated dominant males, and directly, by destroying the eggs of the dominant female [74]. This competition forces dominant (older) females to engage in frequent aggressive displays towards subordinate (younger) females and repeatedly re-lay clutches [74], which entails an additional energetic cost [76]. Hence, in older (dominant) females the cost of maintaining a high reproductive output, even in the presence of competitors, might be traded-off against the maintenance of the energetically costly nervous system [13]. For example, experiments in the fruit fly and the cabbage white butterfly (*Pieris rapae*) have revealed a trade-off between learning performance and competitive ability [77] or female fecundity [78], respectively. In line with this explanation, when analysing long-term reproductive success in dominant babblers, we found that higher cognitive performance was associated with a lower number of fledglings produced per year. However, a larger sample size will be necessary to test whether this negative relationship between cognition and reproduction differs between males and females.

Secondly, babblers show sex differences in dispersal strategies. Females are more likely than males to gain a breeding position by overthrowing a dominant female in a non-natal group [44]. Accordingly, juvenile females are more aggressive than males, and higher female aggressiveness is associated with younger age at dispersal [79]. On the contrary, males are more sedentary and disperse only when search costs are low [80]. It is possible that these sex differences lead to selection on females for higher cognitive performance early in life, even at the expenses of faster cognitive senescence. Indeed, cognitive performance in young females might be crucial to gain access to breeding positions by enabling them to navigate across territories, identify the sex and rank of conspecifics in non-natal groups, and decide when to engage in aggressive displays [81]. Additionally, in babblers the number of immigrant competitors decreases with pair bond tenure, while reproductive success increases [41]. This suggests that on average the risk of losing a breeding position and thus, potentially, the need to maintain high cognitive performance might decrease with female age. Overall, our findings paired with evidence from previous research in babblers suggest that females may be under selection for higher cognitive performance earlier in life despite faster cognitive senescence and/or cognitive senescence may be accelerated by investment in reproduction and breeding competition. However, longitudinal studies are ultimately needed to describe cognitive ageing trajectories in males and females and clarify how individual rank interacts with age to determine cognitive decline.

Since we used a cross-sectional design, we cannot determine whether cognitive performance declined throughout life in females, or whether only females with lower cognitive performance survived until old ages. Therefore, a third potential explanation for the observed sex differences in age-related cognitive decline is that cognitive performance is negatively linked to survival, at least in females. For example in pheasants (*Phasianus colchicus*), survival in the wild was negatively related to reversal learning performance [82]. As most of the birds tested in the present study are still alive to date, we could not perform a survival analysis to examine the relationship between cognitive performance and survival, but this will be necessary to confirm whether the present findings are due to cognitive senescence or reduced survival of smarter females.

We found that individuals with better GCP produced fewer fledglings per year, which is consistent with a trade-off between cognition and reproduction. GCP did not predict the number of fledglings surviving to nutritional independence, which depended instead on the occurrence of droughts during the breeding season, in line with a previous study [83]. It is possible that parental traits influence offspring survival in the nestling stage but not in the post-fledgling stage, where survival may be more strongly influenced by environmental conditions [43]. Therefore, the extent to which cognitive performance may be under negative selection remains to be determined. Cognitive performance might also be simultaneously associated with life-history traits linked to fitness in different directions [84]. For example, while cognitive performance is negatively related to the number of fledglings produced per year by dominant individuals, it might be positively related to the age at which dominance is acquired in the first place. Future comparisons of the age at acquisition of dominance among individuals whose cognition was tested as subordinates will allow us to address this hypothesis.

Overall, we found that individual cognitive performance covaried across tasks, which is consistent with a general cognitive factor, though alternative explanations cannot be excluded. We considered the effect of individual and social attributes and several proxies of motivation on cognitive performance. We found that GCP depended on sex and age, declining with age in females but not males. Older females also tended to fledge more nestlings per year. By analysing over 10 years of breeding data, we show that individuals with lower GCP produced more fledglings per year. Our findings suggest that cognitive performance is traded-off against reproduction, demonstrating that in order to understand how selection acts on cognition we need to consider not only its benefits but also its costs.

## Data Availability

Data are available from the Dryad Digital Repository: https://doi.org/10.5061/dryad.d51c5b06v [85]. Additional information is provided in the electronic supplementary material [86].
